# Consistent financial practices at community sites improve performance and sustainability: A case study of validated practices at a community clinical research site

**DOI:** 10.1017/cts.2026.10736

**Published:** 2026-03-31

**Authors:** Brad Hightower, Daniel Fox

**Affiliations:** 1 Hightower Clinical; 2 Clinical Research Payment Network

**Keywords:** trials, finance, management, best practices, community sites

## Abstract

Community research site finances continue to emerge as substantial topics of concern. Financial management solutions are possible; however, they must be continuously managed and incorporated to realize optimal business quality. The current case study assessed key financial metrics prior to and one year following a clinical research site’s financial review and implementation of improved practices. Financial activity and sustainability metrics were collected and assessed. Staff were interviewed prior to and after the financial review. Results analyzed differences in processes to further elucidate impact and explore best practices. Following review and best practice implementation, the site realized not only an immediate improvement in record management and an increase in revenue realization, but also a substantial increase in 12-month metrics. Fewer visits were performed year-to-year; however, management improvements yielded overall revenue and accrual increases. The site owner and staff members noted a relief and excitement when “Finance is Everybody’s Job” cultures were adopted throughout site departments. Newly established financial processes demonstrated an immediate improvement in site sustainability and, when routinely managed, stabilized monthly finances during lower visit periods. Future studies may assess long-term effects of financial management or analyze each financial component for process refinement and optimization.

## Introduction

Payments are one of the most contentious challenges that community clinical research sites must face during their business operations [1]. For over a decade, clinical trial payments are late as an industry standard, invoiceable items are missed, and financial communications between internal and external stakeholders are scattered and unreliable due to inconsistent communication plans and personnel turnover [2]. The fault, however, cannot be solely on sponsors and CROs but rather on the internal financial management processes of each community research site. It is imperative as a community clinical research site to develop internal financial processes that are fast, accurate, and flexible to meet the needs of its global partners.

## Background

Sponsors and CROs routinely pay late compared to agreed-upon contractual negotiations [3], resulting in mistrust between parties and substantial discouragement from physicians to continue future participation [4]. Promises are broken, physicians avoid further damages, and potential trial participants and future patients risk losing access to new, innovative, and life-saving therapies [5]. The clinical research industry, therefore, harbors broken financial systems that are critically impacting its mission to translate innovative technologies into patient care as quickly, efficiently, and safely as possible [6], [7].

For years now, sites and advocates continually call for financial changes [7]. Sites are not paid according to contractual terms [8], [3] and consequently experience limited operating cash, untimely payment, lack of financial transparency, and significantly underfunded negotiations [9]. Eight years ago, only one in three sites reported having sufficient resources to operate for 90 days [7], which has not improved over time. Further, when sites are paid, it often takes as long as 120–140 days from participant visit to payment received [2].

The industry continues to exist in a financial crisis. Amidst the pandemic in 2020, 31% of sites validated this fragile economic ecosystem as they reported to ACRP a legitimate fear of permanently closing operations due to the pandemic and financial constraints [10]. Health institutions continually find fewer physicians interested in performing research, primarily, according to FDA’s ex-commissioner Robert Califf, “by erosions of system efficiency, waning interest in clinical research among practitioners, and financial insolvency among participants in the clinical research enterprise” [11]. Clinical sites have been frustrated with CRO mismanagement for years and have reported across conference circuits and within inner circles that CRO’s generally tend to ignore contractual obligations and avoid timely payments. These financial disparities frequently threaten a site’s capacity to support quality clinical trials and undermine collaborations between sponsors and sites [12].

As late or no site payments continue to become a standard in our industry [4,8], community research sites must establish thorough financial management processes to ensure they successfully and efficiently retrieve all of the resources they have rightfully earned and hold their industry stakeholders continuously accountable [13].

## Study objectives

The current study elucidates the impact of actively managed financial practices at a community research site. To accomplish its objectives, the study assessed key financial metrics prior to and one year following a clinical research site’s financial review and implementation of improved financial management best practices.

## Financial review methods

**Figure 1.**
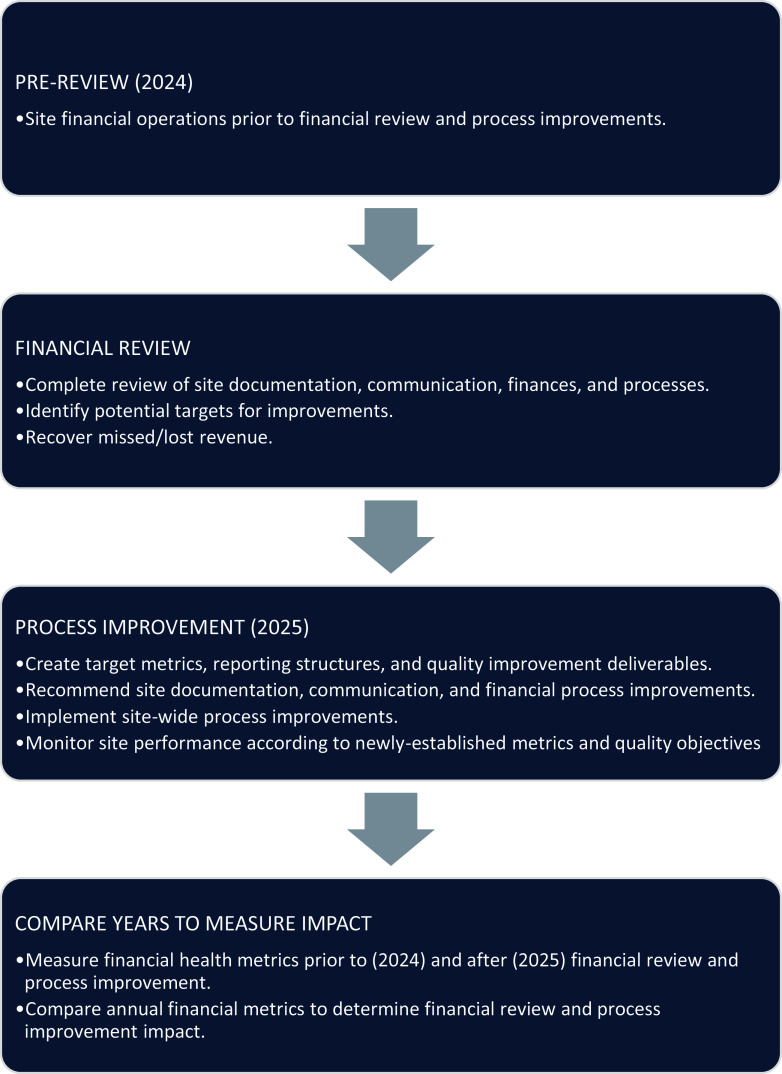
Schema describing study design, milestones, and timelines. A community clinical research site’s financial health metrics were assessed 1 year prior to (2024) and after (2025) a comprehensive financial review and process improvement implementation.

Case study methods were applied prior to (pre-review), during (review), and after (post-review) a comprehensive financial review (Figure 1).

### Timeline

The study timeline ranged from January 2024 to December 2025. Pre-review timelines comprised January 2024 to December 2024. The financial review and initial process improvement ranged from December 2024 to April 2025. Post review and financial assessment was assessed from May 2025 to December 2025.

### Metrics and impact definitions

Each study phase measured holistic impact metrics for overall financial health. Records assessed presence and accessibility of contracts, remittance detail, bank deposit matches, and invoice history by date, value, and department or assignment to the three pillars of a clinical research site, comprising administrative, regulatory, and operations categorizations [13]

- Administrative Pillar: All things contractual, legal, financial, procurement, and business development that support a clinical research site’s infrastructure.
- Regulatory Pillar: All services involving quality assurance (QA) and compliance. Regulatory revenue includes IRB submissions, query resolution and monitoring visits, QA oversight, long-term document storage, etc.
- Operations Pillar: All services involving trial participant engagement and trial performance. Operations revenue includes but is not limited to visits, ancillary services, and procedures.

Cash flow measured monthly deposit values whereas accrual was assessed by value per month, value per visit, and the number of revenue-contributing sponsors per month.

To respect the financial privacy of the site and protect sensitive information, values were redacted and converted into ranges and year-vs-year percentages.

### System access

Access to integral systems were required prior to review including, but not limited to, site e-mail systems, file sharing folders, CTMS applications, third-party payment portals, accounting software systems, and bank systems or deposit records.

### Document retrieval

All necessary documents such as legal (CTAs, amendments, etc.) and financial documents (invoices, remittance detail, AR, aging, etc.) were inventoried and categorized for active trials and trials that were closed within 60 days prior to the financial review. Any trial older than the established timelines were likely not viable for financial reclamation and removed from the study’s scope.

Following document inventory and review, all invoices and bank deposits within the review’s timeline parameters were inventoried and recorded for completeness and accuracy. Finally, remittance detail was compared to bank deposits to identify the percentage of confirmed, documented, and remitted payments throughout the review’s targeted timeline.

### Staff interviews

Staff from all site pillars were interviewed to assess their engagement and awareness of site financial systems, staff opinions about site finances, and the overall financial health of the business.

Personnel were asked similar questions that targeted their engagement with financial departments, an understanding of the overall financial flow of the site, what they feel might be pain points of the financial process, and what they hope will be a result of the financial review.

Following the financial review, staff were interviewed again to follow-up on their original responses and identify any changes in opinion or perceived operational throughput.

## Pre-intervention assessment

Following financial review, assessments and outcomes identified substantial opportunities for improvement and best-practice implementation (Table 1).

**Table 1.** Comparison of practices before and after a comprehensive review of a community site’s financial processes and recommendation for best practice changes. Following financial review, the site adopted new and potential best practices to ensure efficient and thorough financial management

Previous practice (inefficient/high-risk)	New/best practice (reduced-risk)
Critical information sent and stored from individual e-mail addresses without contingency communication plans for site administration.	Critical information sent, stored, and managed by group e-mail addresses and teams.
Invoice systems and tracking within CTMS systems, which fragmented records when CTMS platforms change.	Invoice systems migrated into independent accounting systems for continuity of documentation and records regardless of CTMS platforms used.
Trials entered and remained in CTMS systems as “active” indefinitely – months or years after close out and potentially causing unnecessary and wasteful software expenses.	Just-In-Time trial management in CTMS – Activate only at site activation and deactivate immediately after each trial’s close out visit.
Limited financial communication between site departments and teams.	Continuous and direct communication between finance and site departments. Quick references for invoice triggers and properly-programmed electronic systems.
Third-party sponsor payment portals used as a default, resulting in loss of access to invoice submission processes and payment detail.	Third-party sponsor payment portals declined as much as possible, giving sites control over their own financial processes without a dependence on sponsors for access and invoice control.
Sub-Optimal Negotiations and lost revenue opportunities.	Enhanced negotiation processes, complete financial committee reviews, and contractual provision requirements.
Vague and unidentifiable file names sparsely archived in individual e-mail boxes and unshared folders.	Standardized naming structure for legal and financial documents that are fully archived and accessible for all approved site stakeholders.
Inconsistent to non-existent revenue invoicing and aging cycles.	Continuous monitoring of all visit and invoice revenue, rapid invoice processes, and routine disputes and follow-ups with site and industry stakeholders.

### Trial assessment

Over 100 trials were identified throughout the audit process.

Of these trials:

- 47% – were identified as standard and viable for financial review;
- 7% – required third-party portal access for information retrieval and payment requests;
- 8% – were managed by a third-party vendor;
- 36% – were closed for at least 3 months, were not viable for financial review due to contractual longstop dates, and were immediately deactivated in CTMS platforms to save operational costs that charge per “active” trial.

### Financial system access

Site leaders granted access to all necessary systems including but not limited to CTMS, third-party payment portals, and accounting software. An e-mail address was created to allow for payment representation and file sharing management.

### Document completeness

Document completeness was assessed for all legal contracts, remittance detail, invoice history, and matched bank deposits.

A comprehensive and accessible archive of all trial contracts and legal documents was not readily available during the financial review. Some of the contracts were either not collected, not recorded, stored in partially executed states, or illegible in the fully executed versions. Many of the archived documents were scattered throughout individual e-mail and inaccessible to the site’s executive stakeholders.

### Bank and financial records

Bank deposit records, invoice history, and study remittance advice were assessed from January 1, 2023, to December 31, 2025. Similar to legal documents, a comprehensive and accessible archive of all trial payment detail was not readily available during the financial review. Less than 30% of bank deposits were successfully matched to payment detail and few invoices were remitted in the site’s management systems. In addition – the site had changed trial management systems within this time period and, consequently, did not have a complete and accurate invoice record on file.

### Staff interviews

Staff interviews comprised similar questions to key stakeholders throughout the three pillars of the clinical research site. All personnel provided differing opinions regarding the site’s financial flow and responsibilities; however, the responses resonated toward similar principles:

- Staff noted challenges accessing and changing the information necessary to properly invoice and remit payments, noting potential challenges in how systems were programmed, permissions to edit within the systems, and access to critical banking information for continuous management.
- Operational and regulatory teams acknowledged limited communication plans with financial teams and noted limited to no touch points with financial teams throughout the lifecycle of a clinical trial.
- Some of the operational team assumed finance was simply something the site finance team worked out with the CRO, void of their participation.
- Staff members felt overburdened, overwhelmed, and did not feel they had time to add additional tasks into their daily routine to support financial management with inter-departmental communication.

### Unclaimed revenue

Once complete, the financial review identified over 100 counts of potentially unclaimed revenue totaling over *one million dollars*. Of the unclaimed revenue, reasons for each case of missed revenue were recorded with assigned percentages. Select cases qualified into multiple reasons and created a percentage sum greater than 100%:

- 43% – NOT INVOICED – ADMIN – Contractual obligations, such as management fees, startup, etc. justified payment; however, an invoice was not issued to collect the revenue.
- 29% – NOT INVOICED – REGULATORY – Regulatory services (protocol amendments, SAEs, monitoring visits, etc.) were performed; however, they were not communicated to finance teams or invoiced.
- 21% – NOT INVOICED – OPERATIONS – Trial services (procedures, tests, ancillaries, etc.) were performed; however, they were not communicated to finance teams or invoiced.
- 18% – DATA ENTRY – The Electronic Data Capture (EDC) system was not updated appropriately, and therefore, payment was not processed.
- ∼1% – INVOICE ERROR – Errors on the invoice (such as address, e-mail address, payor, etc.) resulted in the rejection of requested payments.
- ∼1% – REMIT ERROR – Upon review of remittances, payments were identified to be inaccurate compared to the contract, services performed, and issued payments. REMIT ERRORS resulted in disputed payments and adjustments with sponsors and/or CROs.
- 0.2% – ADMIN REVENUE ERROR – Payment was issued by sponsors; however, payment was not matched in bank deposit reports due to paper checks that were mailed and misplaced. Admin revenue errors required a request for reissuance from the sponsor/CRO.

Upon final reconciliation, ∼19% of the identified potential revenue required write offs, accrual adjustments, and an overall loss in site revenue.

## Interventions/proposed best practices

Following the financial review or upon immediate discovery, general best practices were implemented throughout the site’s infrastructure and financial workflow:

- A shared group e-mail was established to ensure finances were not under the purview of a single individual but rather across a group for continuous quality management and monitoring. This e-mail address was entered as the contact e-mail for all future contracts and amendments going forward.
- A shared but controlled network environment was created to ensure complete visibility and access for all necessary stakeholders to critical financial documents such as contracts, amendments, payment details, and reports.
- A shared but controlled network environment was created to ensure complete visibility and traceability of all invoiceable items. To accomplish comprehensive and complete invoice documentation, invoice systems were developed outside of Clinical Trial Management System (CTMS) platforms as CTMS systems are often not controllable by finance teams for optimal invoice generation.
- Site processes benefit greater with the utilization of accounting software specific not only to site revenue cycles but also expense and overall business analytics. If financial accounting systems are utilized, the use of CTMS invoicing systems creates an unnecessary duplication of data entry from one system to the other. In addition, by isolating invoice history from CTMS software, the site may not have to straddle systems or lose records in the event they opt to change CTMS providers.
- A just-in-time (JIT) communication was established by site SOPs to deactivate trials in CTMS systems immediately upon each trial’s close-out visit to prevent unnecessary charges in per-trial costing structures.
- Quick references were distributed to site staff to properly communicate invoiceable item notifications when electronic systems were not programmable to notify finance teams of invoice revenue accrual [13].
- Policies were established to prevent third-party sponsor payment portals when possible as the sponsor’s ability to control site access to sensitive financial information and the ability to send invoices poses a substantial risk to the site’s ability to operate its business. The use of third-party payment portals also induces unnecessary duplication as sites must manage not only their internal financial systems but also external sponsor platforms.
- Negotiation strategies were reassessed to properly fund trial contracts for responsible and quality research performance.
- The site implemented a standardized taxonomy to name critical financial documents for rapid access, clarity, and searchability.
  1. Upon receipt, legal documents were immediately named and filed according to the below nomenclature:

*{SPONSOR} {PROTOCOL ID} PI {PI NAME} Site {SITE #} CTA/A (Amendment # as needed) FE {EXECUTION DATE}*

The nomenclature allows for immediate recognition of each file and applied an additional quality assurance that requires fully-executed dates to name files, which prompts personnel to ensure they have at all times fully-signed and executed legal documents on file.

1. Upon receipt, payment advice and/or remittance detail were immediately named and filed according to the nomenclature below:

*REMIT {$ VALUE} {SPONSOR} {PROTOCOL ID} # {PAYMENT ID} {PAYMENT DATE}*

Utilization of the above remittance taxonomy per file allows site personnel to rapidly match payments with bank deposits without the need to open numerous vague and repetitively named files.

- A routine revenue cycle was established to ensure continuous and diligent financial management [13].
  1. *At the beginning of each month:*
    - Bank deposits from the previous month are documented and recorded.
    - All pending payments with documentation are matched and reconciled against bank deposit records.
    - Sponsors or CROs are contacted to identify and document any unidentified bank deposits.
    - Once all payments are identified and reconciled, performs a complete follow-up with sponsors or CROs regarding late payments, and a late payment (aging) report is distributed to site leadership to escalate internally (site administration) and externally (CRO/sponsor) as needed.
    - New accruals and invoiceable items are identified by team communication and/or CTMS reports, recorded, submitted to sponsors or CROs, and are added to the site’s accrued revenue.
    - Financial reports and data are created to analyze overall site financial health and distributed to site leadership.
  1. *Throughout the month:*
    - Finance teams address sponsor or CRO queries or escalations and resolve payments with respective site pillars.
    - Finance teams collect, rename, and document any incoming remittance detail and save it in a “pending folder” status until the month’s close.
    - Finance teams send invoices as needed or appropriate during start-up, close-out, or regulatory activities (monitoring visits, protocol amendments, etc.).
    - Finance teams maintain a continual communication path with site teams to reinforce and foster a “Finance is Everybody’s Job” culture.

## Intervention/best practice impact

### Documentation

Modified documentation practices yielded almost immediate improvements:

- Prior to assessment, a centralized and complete document was not accessible. Following review and implementation of new processes, 100% of all legal documents, including trial contracts and amendments were completely categorized, renamed, and archived into rapidly retrievable and shared environments.
- Prior to assessment, a centralized repository of payment details were not available. Following review and implementation of new processes, 96% of all payment document filing systems within the targeted time period were reconstructed, categorized, renamed, and archived into rapidly retrievable shared environments. The unretrievable 4% of payment documents resulted from aged sponsor payment portals out of site control that revoked access and prevented retrieval.
- Prior to assessment, less than half of bank deposit matched to payment detail. Following review and implementation of new processes, bank deposits matched at a 98% rate the year prior and 100% match rate from the time of best practice implementation to present. The 2% of unmatched bank deposits resulted from aged sponsor payment portals out of site control that revoked access and prevented match validation.
- Following continuous financial management practices, the site no longer received funds that remain unidentified.
- Prior to assessment, invoice history was not properly documented or tracked in a centralized location. Following review and implementation of new processes, 100% of invoice history spanning multiple CTMS systems and the site’s new independent system was completely reconstructed and accessible in a shared and monitored environment.

### Staff follow-up interviews

Staff interviews and opinions changed substantially before, during, and after the financial review. Post-review and during active financial management, staff and the site owner reported a sense of relief knowing finances are now under control and better-managed, thereby fostering and growing a “Finance is Everybody’s Job” culture throughout all site teams. Personnel frequently engaged with the finance team, asked questions, and voluntarily contributed as a holistic team to the overall information required for successful financial management.

### Financial performance

Financial activity and sustainability metrics were collected and assessed one year before (2024) and after (2025) performing the site’s financial review and implementation of new best practices.

### Accrual

Year-vs-year, accruals demonstrated notable differences before and after the implementation of new financial management processes.

**Figure 2.**
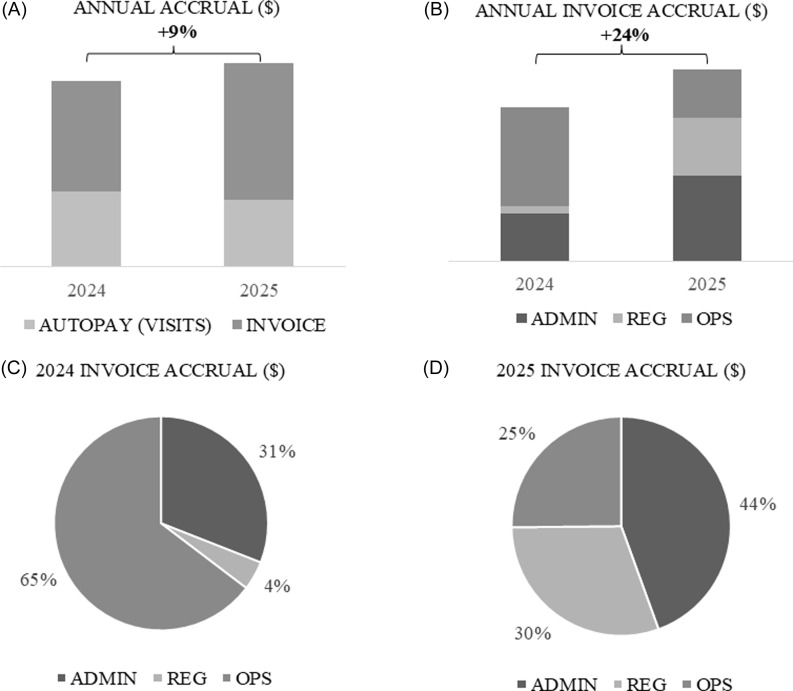
A community research site’s annual autopay vs invoice accrual (A) and invoice accrual by its three pillars (Administrative, regulatory, and operational) (B) one year prior to (2024) and after (2025) a financial review and implementation of new financial management practices. As autopay (visits) decreased, invoice accrual increased; creating a supporting revenue stream during times of low operational revenue accrual. In addition, 2025 regulatory and administrative invoice activity demonstrated continuous and present accrual each month (C, D).

Throughout 2025, autopay accrual (participant visits) remained relatively low; however, the implementation of new financial management practices allowed for the accrual of invoiceable revenue to normalize revenue and compensate for times of low participant visit volumes (Figure 2A) with a notable increase in the number of invoices sent. Two high peaks of accrual in February 2025 and July 2025 comprised invoiceable activity resulting first from the financial review and unrecovered revenue followed by an adjustment to negotiation strategies and therefore an increase in start-up and invoiceable activity for all new trials going forward. Thus, the increased compliment of invoice activity to underlying autopay accrual increased the site’s overall annual accrual.

The year-vs-year invoice mix by pillar noted substantial differences (Figure 2B). Little (4%) regulatory invoice activity was recorded prior to the implementation of new financial processes whereas afterward it recorded a 30% increase in overall regulatory invoicing. Operations, however, recorded reduced invoice activity in 2025 (25%) vs. 2024 (65%). Therefore, although participant visits and operational invoice activity decreased in 2025, the newly realized regulatory and administrative revenue allowed the site an ability to earn similar revenue amounts as it performed fewer visits.

### Visits time value, and active revenue trials

**Figure 3.**
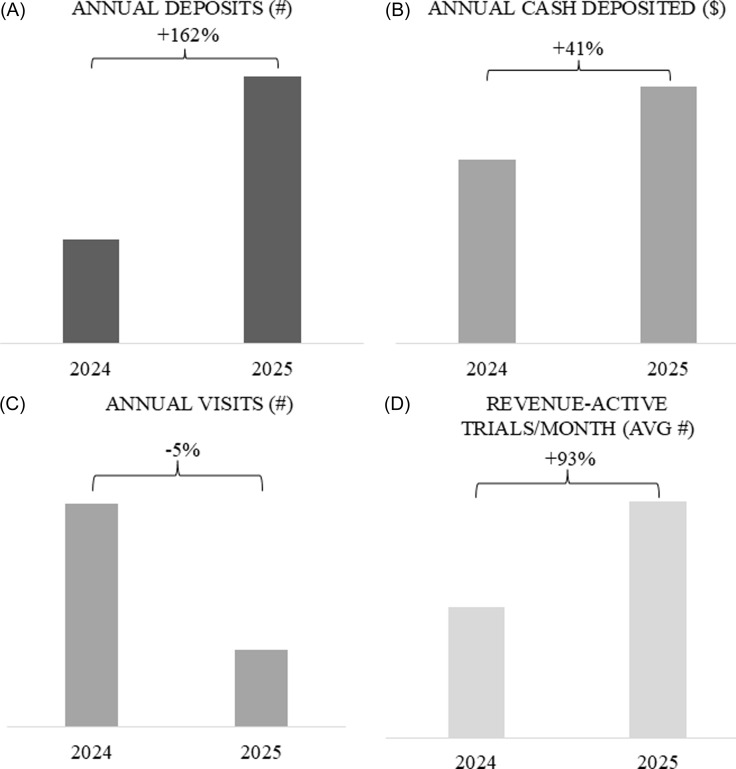
A community research site’s #Annual deposits (A), annual cash deposited (B), #Visits (C), and AVG #Revenue active-trials (D) one year prior to (2024) and after (2025) a financial review and implementation of new practices. Overall visits by month were reduced in 2025 vs. 2024. $/Visit steadily declined as 2025 progressed and # revenue active trials increased in 2025. Deposit frequency increased and contributed to increased cash deposits in 2025.

Revenue increases were not a direct result of increased operational productivity (visits performed). The overall number of visits in 2025 were reduced compared to 2024 (Figure 3C). The final months of 2025 incurred notably high visit counts due to follow-up low-value and high-volume visits; however, the overall year’s visit count was less than the year prior. Revenue-active trials, however, substantially increased as invoice revenue and responsible aging follow-ups continuously engaged with internal and external stakeholders for optimal accrual capture, rapid cash flow, and continuously efficient revenue cycles. Thus – the realized increases in revenue were not a result of visit volume and participant activity but rather thorough and responsible financial management every month throughout lower visit periods.

### Deposits

Frequent and efficient financial processes also demonstrated improved cash deposit values and increased deposit frequencies (Figure 3A). Deposited cash increased in 2025 vs. 2024. To compliment, the number of deposits increased, which is a direct result of actively managed monthly cycles, aging and follow-ups for outstanding revenue owed, and continuous communication plans established between site departments.

## Discussion

How is something as intuitively simple in business settings so challenging for the clinical trials industry to remediate? In an industry as complex and stakeholder-heavy as clinical research, there may be many reasons. To name a few of the most common culprits, the following descriptions and observations are referenced from true stories and real-world experiences that are shared frequently among community research sites.

### No direct accountability for noncompliance

Every clinical trial comprises a negotiated budget and a clinical trial agreement (CTA); however, the promises made when the contract is signed are frequently not honored. As an industry standard, sites report sponsors and CROs often do not adhere to the payment provisions in these contracts. Net30 (monthly) terms may pay sites in 90 days whereas Net90 (quarterly) terms may pay sites in as many as 140 days [14].

Unfortunately, the consequence of neglecting the promises made is minimal. Sponsors refuse to approve the same late fees in US contracts that are required in other countries [15]. A site’s general protections from such non-compliance often escalates to extremes rapidly, such as withholding of data entry or delay of monitoring visits until sponsors can successfully achieve financial compliance.

Site reports of sponsor/CRO financial noncompliance echo throughout the industry, confirming that even now poor site payments continue to plague clinical trial performance [9,16]. While potential solutions are currently under construction [17,18], efforts continue to develop point solutions across niche sectors and specific vendors. To date, a comprehensive and impactful industry-wide solution has yet to gain momentum in the overall industry to eliminate its current culture of acceptable unaccountability and financial non-compliance.

### Improper, unreliable, or no remittance tracking

In the uncommon event sites are paid on time, the challenges have only just begun. Sites report continuous difficulties accessing the itemized and correct remittances they need to properly account for and record payments for services provided. Remittances are sent across the industry through a number of venues and range from helpful and collaborative PDF documents to zero remittances without individual requests, bulk or lump sum remittances that do not align with agreed-upon CTA terminologies or values, required portal logins out of site control for access, and even remittances that are not only incorrect, but also combined with numerous trials and sponsors without defined separation or clear payment descriptions.

Depending on the CRO and sponsor, sites report times to receive a remittance or remittance clarity from 4 weeks to 175 days [9]. In the event of an erroneous remittance, sites have little to no path to dispute.

### Internal site misunderstandings – “It’s not my job”

Far too often, a site’s internal administrative, regulatory, and operations teams create responsibility siloes that do not allow for the flow of financial information communication.

Operations and regulatory teams often claim financial reporting is “not their job” and opt to continue their daily operations without adjusting communication pathways to ensure their site is capturing the revenue owed for each trial. Voluntary siloing throughout each of the industry’s stakeholders and divisions results in an internal inefficiency that costs research sites hundreds of thousands of dollars in unclaimed revenue.

### External communication breaks

“Where’s our payment?”; “Where’s the remittance?”; “This is wrong, why?” Thousands of questions arriving into the e-mail boxes of our global CRO payment specialists. The overwhelming amounts of work result in poor service quality, frustrated stakeholders, and overburdened CRO employees.

Consequentially, the long communication time periods that occur simply to achieve remittance or query resolution lead to extensions in accounts receivable timelines and increased personnel time and cost required to continue following up on easily answered queries.

## Conclusion

Newly established site-level financial processes demonstrated an immediate improvement to site sustainability and, when routinely managed, stabilized site finances with overall increased revenue month after month.

If applied methodically and continuously, these “best practice” financial processes may empower local community sites, strengthen their financial sustainability, and allow them to continue stable and high-quality research performance for their sponsors and trial participants.

Future studies may assess long-term effects of financial management or analyze each financial component for process refinement and expansion into sponsor/CRO perspectives.
